# Enhanced Circadian Clock in MSCs-Based Cytotherapy Ameliorates Age-Related Temporomandibular Joint Condyle Degeneration

**DOI:** 10.3390/ijms221910632

**Published:** 2021-09-30

**Authors:** Sa Cha, Sueng-Min Lee, Jiangyue Wang, Qing Zhao, Ding Bai

**Affiliations:** State Key Laboratory of Oral Diseases & National Clinical Research Center for Oral Diseases, West China School of Stomatology, Sichuan University, Chengdu 610041, China; chasa914@hotmail.com (S.C.); tmdala5547@gmail.com (S.-M.L.); SCUWANGJY@126.com (J.W.)

**Keywords:** temporomandibular joint, mesenchymal stem cell, circadian clock gene, regenerative medicine, aging

## Abstract

Aging has been proven to be one of the major causes of temporomandibular joint (TMJ) disability and pain in older people. Peripheral circadian rhythms play a crucial role in endochondral ossification and chondrogenesis. However, the age-related alterations of circadian clock in TMJ structures are seldom reported. In the current study, TMJ condyles were extracted from young (4-month-old), middle-aged (10-month-old), and old-aged (20-month-old) adults to detect the morphology and circadian oscillation changes in TMJ condyles with aging. The transcriptome profile of *Bmal1*-deleted bone-marrow mesenchymal stem cells (BMSCs) and controls were explored to reveal the circadian-related differences at the molecular level. Furthermore, the reparative effects of *Bmal1*-overexpressed BMSCs-based cytotherapy in aged TMJ condyles were investigated in vitro and in vivo. Aged TMJ condyles displayed damaged tissue structure and an abolished circadian rhythm, accompanied by a progressively decreasing chondrogenesis capability and bone turnover activities. The deletion of *Bmal1* significantly down-regulated chondrogenesis-related genes *Prg4*, *Sox9*, and *Col7a1*. *Bmal1*-overexpressed BMSCs presented improved migration capability ex vivo and attenuated age-related TMJ condylar degeneration in vivo. These data demonstrate the crucial role of circadian timing in the maintenance of osteochondral homeostasis, and indicate the potential clinical prospects of circadian-modified MSCs therapy in tissue regeneration.

## 1. Introduction

Age-related diseases affect millions of individuals and cause heavy economic burdens [1]. As the bilateral synovial articulations bear lifelong vocal function and heavy occlusal loading, the temporomandibular joint (TMJ) condyles undergo progressive degeneration and are frequently involved in osteoarthritis (OA) with aging [2,3]. Aging has been proven to be one of the major causes of joint disability and pain in older people, featured with progressive articular degeneration and loss of normal tissue structures [2]. Age-related TMJ degeneration causes wild changes in condylar cartilage, subchondral bone, synovial fluid and other relative structures [2,4]. However, the detailed mechanism is yet to be unveiled and effective therapeutic approaches are urgently needed.

The mammalian circadian clock dominates the daily (~24 h) rhythms in activities and physiology [5]. The central timekeeping system is located in the suprachiasmatic nucleus (SCN), receiving photic signals and orchestrating the tissue-specific peripheral circadian clock with molecular signals [6]. Peripheral circadian oscillations are cell-autonomous and are synchronized by the SCN outputted central clock. This subtle regulation system is derived from the translational negative feedback loops of heterodimers BMAL1/CLOCK and downstream circadian-controlled genes [7]. Recent studies showed that the circadian clock system had a close relationship with age-related pathways, including mTOR signaling [8], insulin/IGF-1 [9], and PI3K/AKT signaling [10]. Circadian-modifying medicine, time restriction and dietary restriction have been proven to improve the lifespan and healthspan of the circadian system [10,11]. Therefore, the concepts of circadian modified chronotherapy are considered to be the promising antiaging interventions. However, the age-related alterations of circadian clock rhythms and the circadian interventions in treating age-related TMJ condyle degeneration have not been reported before.

Due to the limited self-repairing capability of articular cartilage, conventional therapeutics for TMJ-OA have mainly focused on effective symptom control [3,12]. The mesenchymal stem cells (MSCs) are a group of plastic adherent cells with the capability of self-renewal and multilineage differentiation. Moreover, the feature of immune escape makes it suitable for xenogeneic cell-based tissue engineering, in which strong inflammatory response is absent [13,14]. Therefore, MSCs-based therapy has had added great value after being introduced in regenerative medicine and disease therapeutics [15]. However, the limited migration capability of donor MSCs toward target tissue is still challenging for current MSCs-based regeneration medicine. Recent studies have reported successful genetically modified MSCs-based therapies on several diseases [16,17,18], which improves the migration, survival, and differentiation of donor MSCs and achieves good reparative results. This present evidence highlights the continued interests and novelty in this field.

The aim of this study was to investigate the age-related morphology and molecular and circadian rhythms changes in TMJ condyles and illustrate the potential links and mechanisms between the circadian clock and age-related TMJ degeneration. Using genetic modification, we further applied clock-enhanced MSCs therapy, to develop the pilot concepts of circadian clock-modified tissue regeneration in the treatment of age-related TMJ degeneration.

## 2. Results

### 2.1. Integrity of TMJ Condyle Cartilage and Subchondral Bone Damaged with Aging

**Figure 1:**
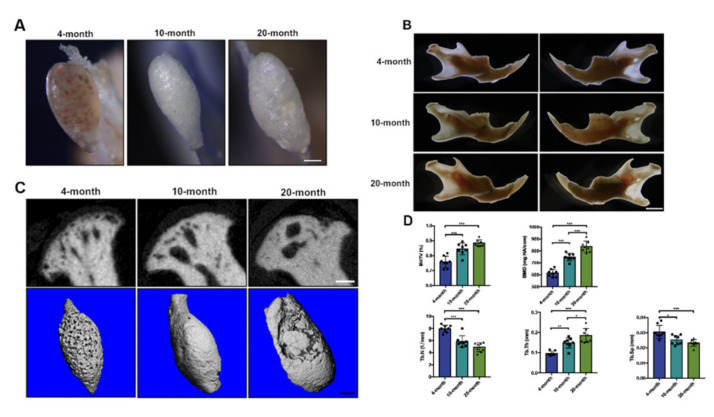
Osteochondral interface and subchondral bone of TMJ condyle gradually ossified with aging. (**A**,**B**) Microscope photographs of the mandibles and TMJ condyles in 4-month-old, 10-month-old, and 20-month-old mice. Scale bar: 200 μm for data A and 1 mm for data B. (**C**) Representative 3D constructed and coronal-sectional images of TMJ condyles in different age groups. Scale bar: 200 μm. (**D**) Quantitative micro-CT analyses on subchondral bone of TMJ condyles in different age groups. (*n* = 8) * *p* < 0.05, ** *p* < 0.01, *** *p* < 0.001.

To identify the age-related morphological change in TMJ condyles, we focused on three specific life phases: mature young adult, middle-aged adult, and old-age adult. According to previous studies that determined the approximately comparable life phases between human beings and mice [19,20], TMJ condyles were isolated from 4-month-old (young adult), 10-month-old (middle-aged adult), and 20-month-old (old-age adult) mice. The microscope photographs of TMJ condyles (Figure 1A,B) showed significant morphology alterations in the geometric shape and condition of the osteochondral surface. The shape of TMJ condyles became longer in the sagittal direction and flatter on the condylar surface with aging, which might result from adaptation remodeling to continuous masticatory force. In the aged mice, the TMJ condylar surface became uneven with irregular erosion, which coincides with the age-related degeneration in human being joints [21]. To further understand the age-related change in the subchondral bone, micro-CTs were performed on each age group. The coronal-section images of the TMJ condyles showed a progressive increase in bone volume and enlarged subchondral cysts in TMJ condyles with aging (Figure 1C). The quantitative analysis of subchondral bone supported the previous finding (Figure 1D). The bone volume/tissue volume ratio (BV/TV) and bone mineral density (BMD) were significantly increased in 20-month-old TMJ condyles, while the trabecular number (Tb.N) and trabecular separation (Tb.Sp) were significantly diminished in aged TMJ condyles and the trabecular thickness (Tb.Th) was increased with aging. Overall, the radiographic manifestations of bone degeneration in aged TMJ condyles were consistent with signs detected in human aged joints.

**Figure 2:**
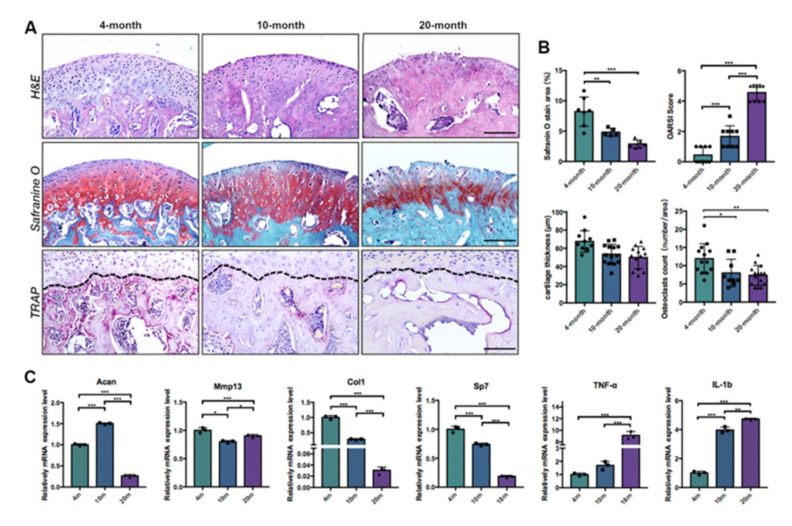
Cartilage degeneration and subchondral bone remodeling decreased in aged TMJ condyle. (**A**) H&E, Safranin O and TRAP staining sagittal sections of the 4-month-old, 10-month-old, and 20-month-old TMJ condyles. Scale bar: 50 μm. The black dotted lines indicated the osteochondral interface of TMJ condyles. (**B**) Quantitative analyses of cartilage thickness, safranin O staining area, OARSI score in safranin O staining sections, and osteoclast counts in TRAP staining sections. (*n* > 6) * *p* < 0.05, ** *p* < 0.01, *** *p* < 0.001. (**C**) qRT-PCR analyses of the mRNA expression of Acan, MMP13, Col I, Sp7, TNF-α, and IL-β of TMJ condyles in different age groups. * *p* < 0.05, ** *p* < 0.01, *** *p* < 0.001.

Next, we sought to examine the histological changes in TMJ condyles with aging. H&E staining showed that the thickness of the proliferative layer zone and hypertrophic layer zone on TMJ condylar cartilage was significantly decreased in middle-aged and old-age mice (Figure 2A,B). Furthermore, the integrity and clear layer structure of cartilage were progressively damaged with aging. Safranin O staining and quantitative analysis revealed an obvious decline in safranin O positive area with the aging, which indicated a significant loss of glycosaminoglycan in the cartilage and subchondral bone in age-related TMJ condyle degeneration. The Osteoarthritis Research Society International (OARSI) histomorphology score of TMJ condyles was higher as the mice grew old. The osteoclasts in the subchondral bone were detected by tartrate-resistant acid phosphatase (TRAP) staining. Quantitative analysis of TRAP staining revealed a significant decline in osteoclasts activities in subchondral bone with aging.

We further examined the age-related molecular changes in the TMJ condyle. The qRT-PCR analysis was performed on the TMJ condyles in different age groups, to determine the expression of chondrogenic, osteogenic and inflammatory markers including *Acan*, *MMP13*, *Col I*, *Sp7*, *TNF-α*, and *IL-6*. The expression of *Acan*, the proteoglycan found in articular cartilage, increased from the young adult to the middle-aged periods and diminished in old age. On the contrary, the expression of matrix metallopeptidase 13 (*MMP13*), the collagenase related to extracellular matrix degeneration, decreased during middle age and was elevated in old age (Figure 1C). The osteogenic markers *Col I* and *Sp7* were significantly declined with aging, while the inflammatory markers *IL-6* and *TNF-α* became prominent in older age. Together with our previous histological finding, a significant decrease in osteogenesis and osteoclastic activities was observed in the subchondral bone of aged TMJ condyles, which indicated that the bone turnover activities in the subchondral bone were flattened with aging.

### 2.2. Clock-Genes Expression and Circadian Molecular Rhythms Were Disrupted in TMJ Condyle Cartilage with Aging

**Figure 3:**
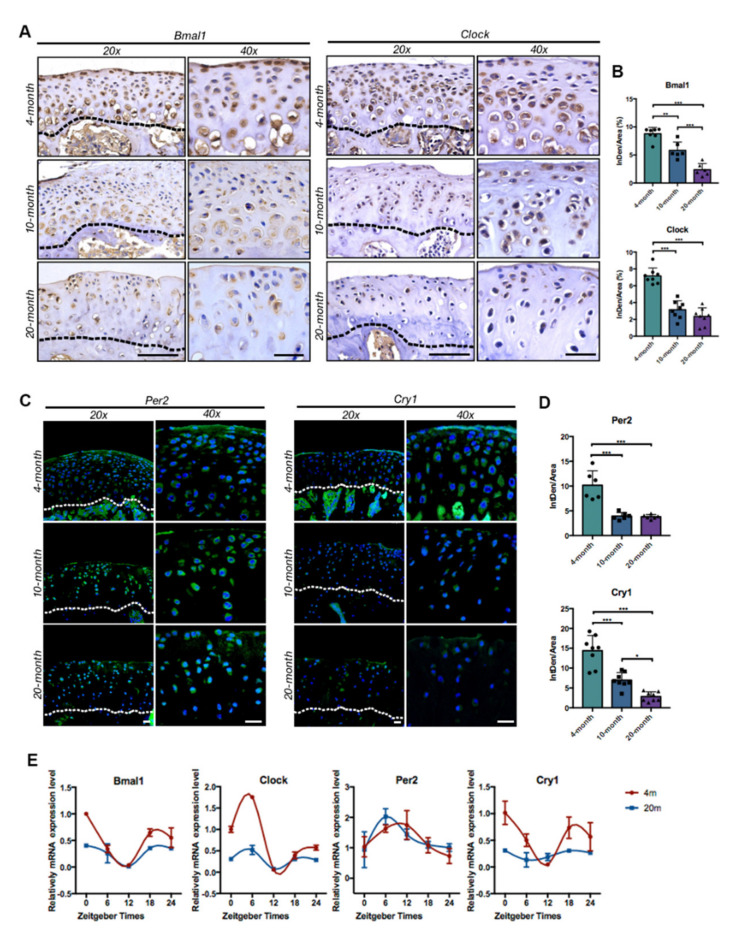
Decreased expression of core circadian clock genes and blunted molecular rhythms of aged TMJ cartilage. (**A**) Representative images of immunostaining for BMAL1 and CLOCK in TMJ condyles in different age groups. Scale bar: 75 μm for 20× and 25 μm for 40×. (**B**) Quantification of percentage of positive staining in data A (*n* = 6–8) * *p* < 0.05, ** *p* < 0.01, *** *p* < 0.001. (**C**) Representative images of immunostaining (fluorescent) for Per2 and Cry1 in TMJ condyles in different age groups. Scale bar: 20 μm. (**D**) Quantification of percentage of positive staining in data C (*n* = 6–8) * *p* < 0.05, ** *p* < 0.01, *** *p* < 0.001. (**E**) qRT-PCR analyses of molecular rhythmic expression of circadian clock genes (*Bmal1*, *Clock*, *Per2*, and *Cry1*) in 4-month-old and 20-month-old TMJ condylar cartilage. The black (or white) dotted lines indicated the osteochondral interface of TMJ condyles.

To investigate the role of circadian genes in age-related TMJ condylar degeneration, immunostaining was performed on condylar sections in different age groups (Figure 3A–D) to detect the expression alterations of core circadian genes. As shown in Figure 3A,B, core clock-gene BMAL1 and CLOCK were widely expressed and gradually decreased in TMJ cartilage with aging, especially on the hypertrophic layer zone and proliferative layer zone. This change was in parallel with the morphologic development of TMJ condyle cartilage with aging, which highlighted the potential crucial role of the circadian clock in age-related TMJ degeneration. PER2 and CRY1 were the typical circadian-controlled genes (CCGs), which were regulated by BMAL1/CLOCK heterodimers and mediated the transcriptional feedback loops system. Immunofluorescent staining showed a significant decrease in PER2 expression in the TMJ condyles of 20-month-old mice with a younger age, while the CRY1 expression substantially declined in the 4-, 10- and 20-month-old mice with aging (Figure 3C,D).

Next, we further examined the circadian rhythmic expression in TMJ condylar cartilage in young and old-age adult mice. TMJ condylar cartilage at 4 months old and 20 months old was harvested at 6 h intervals according to zeitgeber time (ZT0, ZT6, ZT12, ZT18, ZT24), and qRT-PCR was performed to detect the rhythmic expression of core clock genes. The significantly blunted circadian rhythms of *Bmal1*, *Clock*, and *Cry1* expression were observed in the old group compared with the mature adult group (Figure 3E). The molecular rhythmic expression of *Per2* presented obvious postponement.

### 2.3. Bmal1 Contributes to Osteochondral Development and Chondrogenic Differentiation

**Figure 4:**
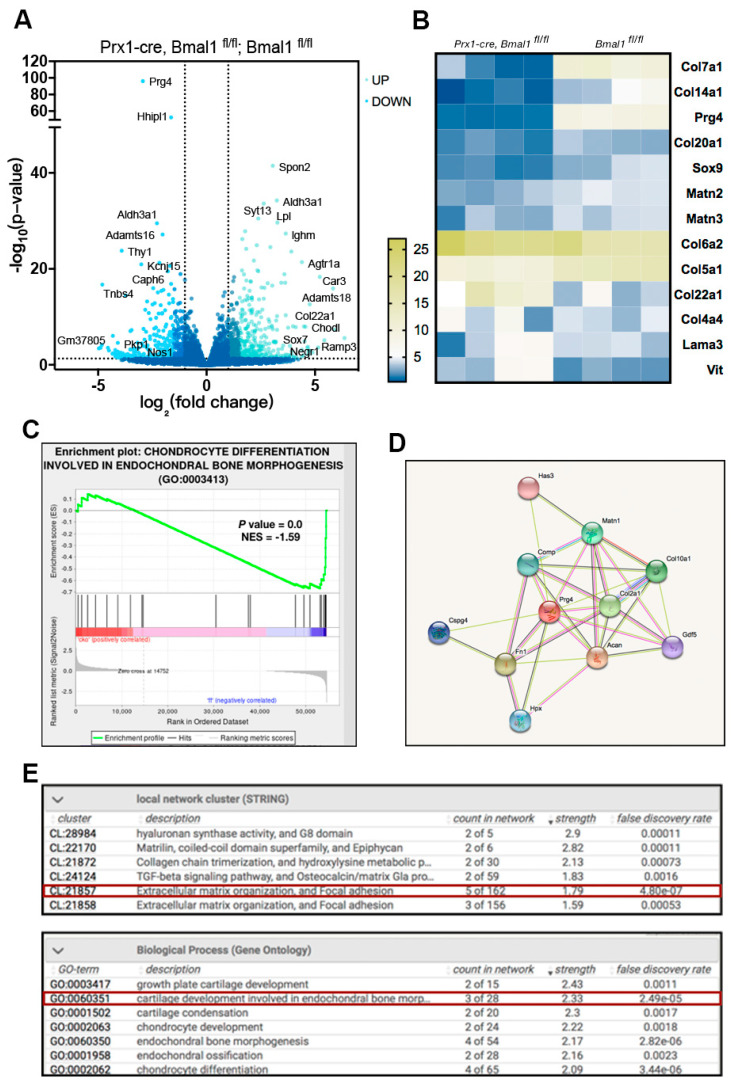
*Bmal1*-deficent BMSCs damaged chondrocyte differentiation and extracellular matrix metabolism. (**A**) Volcano map of differential expressed mRNAs in *Bmal1^fl/fl^* and *Prx1-cre; Bmal1^fl/fl^*, Prg4 was the most significantly down-regulated gene. (**B**) Heatmap of representative chondrogenesis-associated genes (expressed as fold-change). (**C**) GSEA showed decreased enrichment on chondrocyte differentiation involved in endochondral bone morphogenesis in *Bmal1*-deficient BMSCs. (**D**) The protein–protein interaction network of PRG4 using STRING. (**E**) PRG4-associated local network cluster (STRING) and biological process (Gene Ontology); red box indicates the most relevant functional process.

*Bmal1*, as the core circadian gene, has been reported to orchestrate the multiple differentiation of mesenchymal stem cells. In another work, we generated *Bmal1* conditional knockout mice (*Prx1-cre; Bmal1^fl/fl^*) from early limb bud mesenchyme and carried out RNA sequencing (RNA-seq) to examine the expression profile of BMSCs isolated from 4-month-old *Prx1-cre, Bmal1^fl/fl^* and *Bmal1^fl/fl^* mice. The volcano map showed the most obvious differentially expressed genes from *Prx1-cre, Bmal1^fl/fl^* BMSCs, and *Bmal1^fl/fl^* controls, from which the *Prg4* was the most significant down-regulated one by *Bmal1* deletion (Figure 4A). PRG4 is a large proteoglycan which synthesized by cells located at the surface of articular cartilage and is involved in elastic absorption and energy exchange of synovial fluid. Moreover, the expression heatmap further confirmed the down-regulation of chondrogenic marker genes, such as *Col7a1*, *Col14a1*, *Matns2* and *Matns3* (Figure 4B). We also performed a gene set enrichment analysis (GSEA) on our sequence data, which showed a global down-regulation of chondrogenic differentiation genes (Figure 4C). The protein–protein interaction network visualization using STRING revealed that the PRG4-related function might have a strong bearing on chondrogenesis, and the local network cluster and associated biological process analysis reported close links with extracellular matrix organization and cartilage development (Figure 4D,E). This result suggested that *Prg4* and chondrogenesis-related genes were the potential downstream targets of *Bmal1*-mediated osteochondral development and chondrogenic differentiation.

### 2.4. Reparative Effects of Local Transplantation of Circadian Clock Enhanced MSCs on Aged TMJ Region

**Figure 5:**
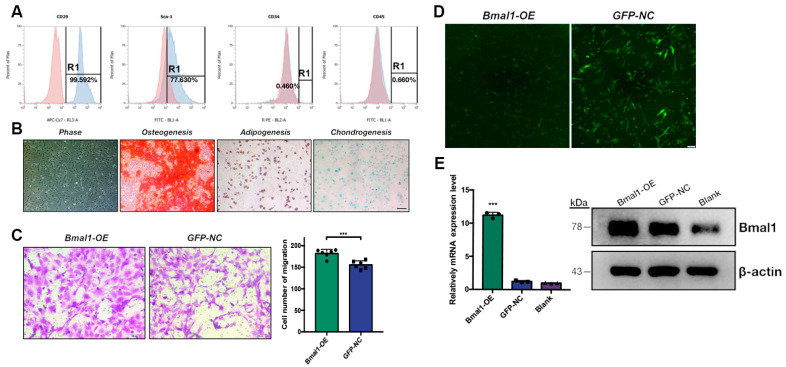
Effect of *Bmal1* overexpression in primary BMSCs. (**A**) Identification of primary BMSCs by flow cytometric analysis. Cells were labeled with CD29 and Sca-1 as positive markers; CD34 and CD45 as negative markers. (**B**) Morphology of cultured BMSCs and Alizarin Red, Oil Red, and Alcain Blue staining of BMSCs under conditional culture medium. (**C**) Fluorescence microscopy of *Bmal1*-OE and GFP-NC BMSCs after lentivirus transfection. Scale bar: 200 μm. (**D**) qRT-PCR and Western blot analysis of *Bmal1* expression of *Bmal1*-OE and GFP-NC BMSCs. *** *p* < 0.001. (**E**) Cell migration of *Bmal1*-OE and GFP-NC BMSCs was determined by transwell assay. *** *p* < 0.001.

Since age-related TMJ condyle degeneration was strongly linked to *Bmal1* depletion on articular cartilage, we next examined the reparative effects of circadian-enhanced MSCs transplantation on the aged TMJ region. Primary BMSCs were flushed out from the femur and tibia of 2-month-old mice and cultured as previously described [22]. Flow cytometry analysis showed that the isolated cells were positive for BMSCs surface markers CD29 and Sca-1, and a lack of hematopoietic stem cells surface markers CD45 and CD34. The spindle- and triangular-shaped BMSCs at passage 2 were observed under phase contrast microscopy and multiple differentiation capability was proved by Alizarin Red staining, Oil Red staining and Alcain Blue staining after conditional medium induction (Figure 5A,B). BMSCs overexpressing *Bmal1* (*Bmal1*-OE) and GFP control (GFP-NC) were constructed by lentivirus transfection. The transfection efficiencies were visualized under fluorescence microscopy and determined by qRT-PCR and Western blot, as shown in Figure 5C,D. The original Western blot acquisitions for cropped images were shown in Figure S2. The migration capability of donor cells into the target tissue is an essential condition in MSCs-based therapies. Therefore, transwell assays were performed to investigate the effects of *Bmal1* overexpression on MSCs migration. As shown in Figure 5E, the number of migrated BMSCs was significantly increased in *Bmal1*-OE in the control group. This result revealed that BMSCs overexpressing *Bmal1* presented greater migration capability than GFP-NC.

**Figure 6:**
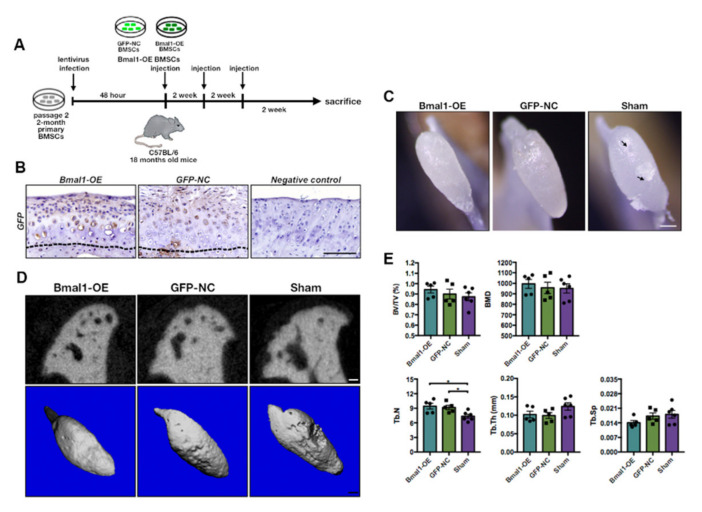
Delivery of *Bmal1*-OE and GFP-NC BMSCs into aged mandibular TMJ region. (**A**) Workflow chart diagram of the *Bmal1*-OE and GFP-NC BMSCs transplantation process. (**B**) GFP immunohistochemistry staining showing GFP-positive cells implanted in the TMJ condylar cartilage. The black dotted lines indicated the osteochondral interface of TMJ condyles. (**C**) Microscope photographs showing the reparative effect of *Bmal1*-OE BMSCs delivery on the mandible and TMJ condyles. The black arrows indicate the uneven surface of the articular surface. The black arrows indicated pits on TMJ condylar surface. (**D**) Representative 3D constructed and coronal-sectional images of TMJ condyles in different groups. Scale bar: 200 μm. (E) Quantitative micro-CT analyses of subchondral bone of TMJ condyles in different groups. (*n* = 5–6) * *p* < 0.05.

**Figure 7:**
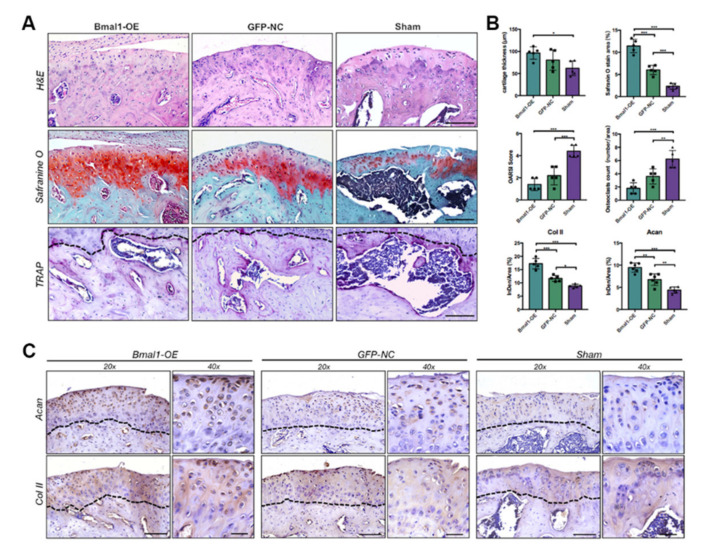
Transplantation of *Bmal1*-OE BMSCs reversed age-related TMJ condylar cartilage degeneration and subchondral bone loss. (**A**) H&E, Safranin O, and TRAP staining of sagittal sections of the *Bmal1*-OE, GFP-NC, and Sham group TMJ condyles. Scale bar: 50 μm. (**B**) Quantitative analyses of cartilage thickness, safranin O staining area, OARSI score in safranin O staining sections and osteoclasts counts in TRAP staining sections; quantitative analyses of immunostaining for ACAN and COL II in TMJ condyles in different groups. (*n* = 5–6) * *p* < 0.05, ** *p* < 0.01, *** *p* < 0.001. (**C**) Representative images of immunostaining for Acan and Col II in TMJ condyles in different groups. Scale bar: 75 μm for 20× and 25 μm for 40×. The black dotted lines indicated the osteochondral interface of TMJ condyles.

The workflow diagram of the MSCs therapy is shown in Figure 6A. To verify the successful transplantation of donor cells, the immunostaining analysis revealed that the implanted GFP-positive BMSCs in aged TMJ cartilage were differentiated into chondrocyte-like cells in vivo (Figure 6B). The microscope photographs of the treated TMJ condyles showed that both *Bmal1*-OE and GFP-NC MSCs-treated TMJ condyles presented a smoother osteochondral surface than the sham group, indicating the restoration of degenerated cartilages after cytotherapy (Figure 7C). The representative micro-CT images further showed an obvious decline in osteophytes around the margin of the osteochondral surface and lessening of subchondral cysts below the articular surface. The micro-CT analysis of the MSCs-treated subchondral bone showed slight but not significant increase in BV/TV and BMD with the sham group, accompanied with an obvious rise in Tb. N and decrease in Tb. Sp and Tb. Th (Figure 6D,E).

Histological staining and quantitative analysis of the condylar sections further revealed that *Bmal1*-enhanced MSCs-treated TMJ condyles were increased in cartilage thickness and decreased in numbers of excessive subchondral cysts. Safranin O-positive areas and OARSI scores revealed a significant recovery of proteoglycan loss in the condylar surface after *Bmal1*-enhanced MSCs treatment. The TRAP staining and quantitative analysis of osteoclast numbers showed a decrease in osteoclast activities in *Bmal1*-overexpressed MSCs-treated TMJ condyle, indicating the regenerative effects of circadian-enhanced therapy in the subchondral bone. The immunostaining and quantitative analysis of the expression levels of chondrogenesis-related markers ACAN and COL II further revealed that more active chondrogenesis was shown in the cartilage layer in the *Bmal1*-OE group than in the control groups. These results imply that transplantation of *Bmal1*-overexpressed BMSCs in aged TMJ cavity could effectively ameliorate age-related cartilage degeneration.

## 3. Discussion

Aging has been proven to be one of the major causes of joint disability and pain in older people. Age-related manifestations in the articular cartilage of knees and hips have been demonstrated in previous studies [23,24]. The temporomandibular joint (TMJ), as a lifelong weight-bearing joint which is frequently involved in osteoarthritis, is seldom reported with age-related morphological and molecular changes. To evaluate the age-related degeneration of TMJ condyles and how this imitates the aging process in human beings, we extracted mice TMJ condyles from 4-month-old, 10-month-old, and 20-month-old mice, which parallels young, middle-aged, and old-age individuals according to humans’ life span. In this study, we observed morphological and molecular changes in the TMJ cartilage and subchondral bone with aging. The following manifestations were recognized as signs of human articular bone degeneration on a quantified scale [21,25]: (a) erosion, (b) generalized sclerosis, (c) osteophytes, and (d) subchondral cysts, which matched our morphologic findings in mice. Furthermore, the 3D reconstructed images and histological analysis of the osteochondral surfaces on TMJ condyles demonstrated the dynamic process of cartilage breakdown with aging. The condylar cartilage was loosened and poriferous in young adults, and became gradually ossified and smooth in middle age, and finally lost its normal structure in old age. In this aging process, the chondrogenesis activities of condylar cartilage increased from young adult to middle age and gradually declined with aging. In parallel, the subchondral bone turnover activities progressively declined with aging, which performed as the decreased activities of osteogenesis and osteoclasts. These findings revealed that the tendency of chondrogenesis and subchondral bone turnover activities on TMJ condylar were different in the aging process.

The peripheral circadian clock has been proven to coordinate various biological processes and has gained increasing interest in the latest decade [26,27,28]. *Bmal1*, as the core circadian clock gene, has been proven to be involved in the limb endochondral ossification during postnatal skeletogenesis [29,30]. Dudek et al. first identified the high expression of BMAL1 in human OA cartilage and demonstrated that the loss of BMAL1 harmed cartilage homeostasis by reducing the expression levels of phosphorylated SMAD2/3 and NFATC2 [31]. Yu et al. reported that *Bmal1* targeted hedgehog signaling and downstream cascades to regulate chondrogenesis and endochondral ossification on mandibular condyles [30]. In addition, the direct links of aging and circadian rhythms in a tissue-specific manner have been well established in previous studies [32,33,34], that have highlighted a potential role of the peripheral circadian clock system in the maintenance of osteochondral homeostasis on the aging of TMJ condyles. Previous studies proved that high-amplitude circadian rhythms could prolong the lifespan and relive symptoms of age-related diseases in several animal models [35,36]. This evidence shows a promising future for enhanced clock chronotherapy on bone and cartilage diseases. In this study, we first investigated the age-related molecular alterations in TMJ condyles and revealed the blunted circadian rhythms and decreased expression of clock genes in TMJ condyles with aging. These findings matched the age-related molecular circadian rhythms alterations in BMSCs in our previous findings [37], and became the fundamental basis of the circadian clock enhanced cytotherapy.

In the past few years, several MSCs-based preclinical applications and pilot clinical trials have been conducted in hip and knee joints [12]. MSCs have been successfully isolated from a variety of tissues such as adipose tissue, umbilical cord, and dental pulp [38]. However, the safety, efficacy and feasibility of these MSCs in cartilage regeneration have been rigorously examined. Dang et al. recently demonstrated that an advanced therapeutic application combining adipose tissue-derived mesenchymal stem cells and dermal-derived collagen could promote cartilage regeneration [39]. Recent studies reported that repetitive injections of exogenous BMSCs could relieve cartilage degeneration in temporomandibular joint osteoarthritis (TMJ-OA) in rodent models [16,40]. However, the effective migration of donor cells toward the target tissue and chondrogenic differentiation still hinder the application of MSCs-based therapy on the TMJ region [18,40]. Lu et al. reported that multiple-time injections of GFP-BMSCs into the TMJ region rescued cartilage degeneration in mice TMJ-OA [18]. In this study, we constructed circadian clock enhanced MSCs by overexpressing the core circadian clock gene *Bmal1*(*Bmal1*-OE). The elevated *Bmal1* expression level and migration capability of *Bmal1*-OE MSCs were confirmed ex vivo. Due to the narrow space of the TMJ cavity in mice, great difficulty has been issued on the direct puncture of the TMJ cavity [41]. Here, we utilized the zygomatic process approached anterosuperior puncture technique (ASPT), which has been reported to have high success rates in previous studies [41,42,43]. The successful transplantation of GFP-labeled BMSCs into the TMJ region were confirmed by immunostaining. The in vivo transplantation was applied with a three-time injection in two-week intervals. Significant reparative effects between the BMSCs-treated TMJ condyles and the sham group were observed by radiographic and histological analysis, from which the circadian-enhanced BMSCs-injected TMJ condyles presented greater cartilage regeneration and matrix deposits.

Recent studies have shown that the peripheral circadian clock system might play an important role in the proliferation and differentiation of MSCs [44]. Our previous work has demonstrated that *Bmal1* promoted BMSCs osteogenesis via activation of Wnt signaling [45,46]. Aside from the negative feedback regulation mechanism, Zhuo et al. reported that *Bmal1* and *Per2* have a synergistic effect on osteoblastic differentiation of BMSCs [47]. In this study, we performed the RNA-seq on *Prx1-cre; Bmal1^fl/fl^* and control BMSCs. A significantly decreased expression level of chondrogenesis genes was observed in *Bmal1*-deleted BMSCs. These results might partly explain the mechanism behind the reparative effect of circadian clock-enhanced MSCs therapy as the donor BMSCs differentiated into chondrocyte-like cells and contributed to the reconstruction of TMJ cartilage. What is worth noting is that the proteoglycan4 (*Prg4*) was one of the most significantly decreased genes in the BMSCs of *Bmal1* deletion. *Prg4* is a proteoglycan formed by cells located in superficial zone of articular cartilage, where *Bmal1* is also highly expressed. This lubricin functioned as the boundary lubricant of articular cartilages and contributed to the energy metabolism in synovial fluid [48]. Therefore, the reparative effects of *Bmal1*-OE BMSCs transplantation might also rely on PRG4-mediated substance exchange in the synovial fluid and the replenishment of the extracellular matrix. Other mechanisms might be involved in the circadian clock-enhanced MSCs therapy that need further investigation.

In summary, our study has summarized the age-related morphologic, histologic, molecular, and circadian rhythms changes in TMJ condyles. Shown by RNA-seq data, the chondrogenesis-related genes and signaling were in regulation of the core circadian clock gene *Bmal1*. Overexpression of *Bmal1* in BMSCs promoted cell migration ex vivo, and transplantation of clock-enhanced BMSCs in the TMJ cavity showed good regenerative effects on articular cartilage and reduced the subchondral bone cysts. This pilot application established the concept of clock-modified therapy for TMJ regeneration and showed good future prospects.

## 4. Materials and Methods

### 4.1. Animal

Male C57BL/6 mice (4-month-old, 10-month-old and 18~20-month-old) were purchased from Chengdu Dossy Biological Technology Co. Ltd. and housed in West China Hospital Animal Center under standard animal housing conditions with a 12 h light and 12 h night cycle. For the analysis of age-related changes in temporomandibular joints, animals were randomly selected and sacrificed in each age group (*n* = 8–14 per group). All procedures and management of the animals were approved by the animal ethics committee of West China School of Stomatology, Sichuan University and the State Key Laboratory of Oral Disease (WCHSIRB-D-2017-046).

### 4.2. Micro-CT Analyses

The temporomandibular condyles were harvested and immediately fixed in 4% paraformaldehyde at 4 °C for 72 h. Collected samples were scanned using a micro-CT scanner (μCT50;SCANO, Switzerland) at a voltage of 70 kVp, a current of 200 μA, and resolution of 4.0 μm/pixel. For the analysis of subchondral bone, a semi-arched region of interest (ROI) was defined at 100μm below the osteochondral interface of the temporomandibular condyle head. The diagram of the selected ROI is shown in Supplementary Figure S1. The bone volume fraction (BV/TV), trabecular number (Tb.N), trabecular thickness (Tb. Th) and trabecular separation (Tb.Sp) were calculated. 

### 4.3. Histological and Immunostaining Analysis 

Following micro-CT scanning, the samples were decalcified in PBS-buffered 10% EDTA (pH 7.4) for 14 days. The decalcified samples were processed, embedded in paraffin, and dissected into 5 μm-thick sections at the sagittal plane. Hematoxylin and eosin (H&E), safranin O staining, and tartrate-resistant acid phosphatase (TRAP) staining were performed according to the manufacturer’s instructions as in the previous study. The Osteoarthritis Research Society International (OARSI) scores were used to grade the cartilage degeneration of temporomandibular joint condyle [49].

Immunostaining was performed as previously described. For IHC staining, the anti-rabbit/mouse immunohistochemical secondary antibody kit (Absin Bioscience) was used following the manufacturer’s instruction. The following antibodies were used: Bmal1 (1:250, #NB100-2288, Novusbio), Clock (1:100, #ab3517, Abcam), GFP (1:100, #NB600-597, Novusbio), Acan (1:200, #13880-1-AP, Proteintech), Collagen II (1:100, #ab34712, Abcam). For IF staining, the following primary antibodies were used: Per2 (1:200, #180655, Abcam), Cry1 (1:200, #ab104736, Abcam). The sections were stained with DAPI to detect the nuclei. After mounting, the sections were photographed with a Leica DM2500 microscope (Leica, Germany). The immunoreactivity of each group was quantified in five different samples and three random areas using ImageJ software.

### 4.4. RNA Extraction and RT-qPCR

To determinate the rhythmic expression of circadian clock genes in 4-month-old and 20-month-old temporomandibular condylar, the total RNA of the temporomandibular condylar cartilage was extracted using TRIzol (Invitrogen) at Zeitgeber Time in 6 h intervals (ZT0, ZT6, ZT12, ZT18, ZT24) according to the manufacturer’s instruction. The RNA concentration was measured with the NanoDrop 2000 (Thermo Fisher Scientific) and reverse transcribed into cDNA using the PrimeScript RT Reagent Kit with gDNA Eraser (Takara). RT-qPCR was performed using SYBR Premix Ex Taq II (Takara) on an Applied Biosystems Quant Studio 7 (Thermo). Relative genes expression was performed by *Gadph* for mRNA expression using the 2^−ΔΔCt^ method. The primers of target genes are listed in Supplementary Table S1.

### 4.5. Bioinformation and Data Analysis

The total RNA samples were extracted using Trizol reagent. A sequencing library was constructed using NEBNext Ultra RNA Library Prep Kit for Illumina (NEB, USA) following the manufacturer’s recommendations, and was then performed on the Illumina HiSeq 2500. The FastQC and FASTX toolkits were used to control the sequencing quality. Sequencing data were mapped to Mus musculus reference genomes by HISATS, and differential expression analysis was performed by Ballgown software. Genes were considered significantly differentially expressed if fold change ≥2.0 and *p* value < 0.05.

The GSEA was performed with GSEA software (http://www.broad.mit.edu/GSEA accessed on 23April 2021) using a sequencing-derived gene list. The protein–protein interaction (ppi) network analysis was performed with STRING (https://www.string-db.org accessed on 16 July 2021).

### 4.6. Cell Culture and BMSCs Characterization

Primary BMSCs were isolated by flushing the bone marrow from the femurs and tibiae of 2-month-old mice as previously described [22,50]. Cells were cultured in α-MEM medium (Hyclone) supplemented with 10% fetal bovine serum (Gibco) and 1% penicillin and streptomycin (HyClone) at 37 °C and in a 5% CO_2_ atmosphere. Cells at passage 2 were used to characterize BMSCs surface biomarkers. The flow cytometric analysis was performed as previously described. The following antibodies were used for FACS analysis: APC-Cyanine7 anti-mouse CD29 (#102225, Biolegend), PE anti-mouse CD34 (#119307, Biolegend), FITC anti-mouse CD45 (#10307, Biolegend), and FITC anti-mouse Sca-1 (#108105, Biolegend).

### 4.7. Lentivirus Transfection

The lentivirus vectors Lv-GFP-Bmal1 and Lv-GFP-mock were purchased from Hanbio Co. Ltd., Shanghai, China. Prior to lentivirus transfection, primary BMSCs at passage 2 were seeded in 6-well plates. Cells were transfected with lentivirus vectors when cells confluence reached 40–50%, and experiments were performed 48 h after infection. The transfected cells were named *Bmal1*-OE and GFP-NC.

### 4.8. Western Blot Analyses 

Cells were lysed in RIPA buffer (Sabbiotech). The protein concentrations were evaluated using a BCA protein assay kit (Beyotime). Equal amounts of proteins in each sample were separated by SDS-PAGE (Bio-Rad Laboratories), transferred to PVDF membranes (Millipore), and blocked in 5% milk. Following incubation with rabbit anti-BMAL1 (1:1000, #14020, Abcam), anti-beta-actin (1:1000, #8457S, CST) at 4 °C overnight, and horseradish peroxidase-conjugated secondary antibody (1:5000, #L3012-2, Sabbiotech), the target protein was exposed using a chemiluminescence kit (Bio-Rad Laboratories).

### 4.9. Migration Assay

Cell migration assays using a transwell system (Corning, 3422) were performed as previously described [51]. Briefly, the lentivirus transfected BMSCs were seeded in the upper chamber at a density of 2 × 10^5^ cells/100 uL/well in a serum-free medium. The lower chamber was filled with a complete medium containing 10% FBS. Following the 36 h of incubation, the migrated cells on the lower chamber were fixed in 4% paraformaldehyde and stained with crystal violet. The number of migrated cells was counted under camera.

### 4.10. Transplantation of Circadian Enhanced BMSCs

We investigated the treatment effect of circadian enhanced BMSCs on age-related TMJ degeneration. The 5000 *Bmal1*-OE and GFP-NC BMSCs were suspended in 20 μL phosphate-buffered saline (PBS) 48 h after lentivirus transfection and injected into the TMJ region of 18-month-old mice (*n* = 5–6) [18]. The aged mice were anesthetized by intraperitoneal injection of Ketamine and Xylazine (100 mg/mL, 2:1, 1 mL/kg body weight). The delivery of cells into the bilateral TMJ regions were performed as previously described [18,39]. Briefly, the mice were laid sidelong after deep anesthesia. We used 30-gauge needles in the most prominent bulge of the zygomatic arch, and along the superomedial of the zygomatic arch toward the TMJ region. Transfected BMSCs were injected into the bilateral TMJ region, respectively, at 0, 2, and 4 weeks successively. In the sham group, 20 μL PBS was injected into the TMJ region.

### 4.11. Statistical Analysis

All data were presented as mean ± SD. Statistical differences of multiple comparisons were determined by one-way ANOVA followed by Tukey’s post hoc test with GraphPad Prism 7 software. The *p* value < 0.05 was considered to be statistically significant.

## 5. Conclusions

In the present study, we have shown that the aged TMJ condyles performed degenerated cartilage, uncoupled bone remodeling, and abolished circadian rhythm. Deletion of the core circadian clock-gene *Bmal1* significantly attenuated the chondrogenic capability in BMSCs. Furthermore, *Bmal1*-overexpressed BMSCs exhibited increased migration capability and improved reparative effects in TMJ condylar cartilage regeneration. These findings highlight the potential important role of circadian timing in maintenance of cartilage integrity, and develop a pilot concept of chronotherapy in cartilage-related regeneration medicine.

## Data Availability

The datasets used and/or analyzed during the current study are available from the corresponding author on reasonable request.

## Supplementary Materials

The following are available online at https://www.mdpi.com/article/10.3390/ijms221910632/s1.
