# Supplementary material for: Enhanced Circadian Clock in MSCs-Based Cytotherapy Ameliorates Age-Related Temporomandibular Joint Condyle Degeneration
Source: Int J Mol Sci. 2021 Sep 30;22(19):10632. doi: 10.3390/ijms221910632 (PMC8508754; doi:10.3390/ijms221910632)
Supplement: Supplementary file 1 [file ijms-22-10632-s001.zip › Supplementary figure legends.pdf]

Supplementary figure legends

Figure S1 Diagram of selected region of interest in micro-CT analysis

(A) The schematics semi-arched ROI in subchondral bone, which defined at 100μm below the osteochondral interface of the temporomandibular condyle head. (B) The system operation interface of defining the region of interest.

Figure S2 Full western blot acquisitions for cropped images in Figure 5
