# Supplementary material for: Enhanced Circadian Clock in MSCs-Based Cytotherapy Ameliorates Age-Related Temporomandibular Joint Condyle Degeneration
Source: Int J Mol Sci. 2021 Sep 30;22(19):10632. doi: 10.3390/ijms221910632 (PMC8508754; doi:10.3390/ijms221910632)
Supplement: Supplementary file 1 [file ijms-22-10632-s001.zip › Table s1.pdf]

Table S1. List of primers used for qRT-PCR in this study.

Genes	Upstream (5'-3')	Downstream (5'-3')
Gapdh	ACCCAGAAGACTG TGGATGG	CACATTGGGGGTAGG AACAC
Bmal1	AACCTTCCCGCAGC TAACAG	AGAGGACCAGGGGAC AT
Clock	AGGGCTGAAAGAC GGCGAGAAC	GTCGGTGTGGAGGAA GGGTCTGA
Per2	AGAACGCGGATAT GTTTGCTG	ATCTAAGCCGCTGCA CACACT
Cry1	CCCAGGCTTTTCAA GGAATGGAACA	TCTCATCATGGTCATC AGACAGAGG
Acan	CCCCTTCGATAGTC CTGTCATTC	CGCCACTTTCATGACC GAGAGAC
MMP13	CGGTGTCAGAGTCT AGGGGA	ATCACCAGGATTGGA CATGG
Col I	GGCCAAAGGAGAAA CCAGGAAG	GGGCAGAGGCAGTCA GGAGCT
Sp7	ATGGCGTCCTCTCTG CTTG	TGAAAGGTCAGCGTAT GGCTT
IL-6	TGATGCACTTGCAG AAAACA	ACCAGAGGAAATTTTC AATAGGC
TNF-α	CCTGGTTGGCTGCTT GCTT	GACTAGCCAGGAGGG AGAACAGA
