# Supplementary figures and images for: Enhanced Circadian Clock in MSCs-Based Cytotherapy Ameliorates Age-Related Temporomandibular Joint Condyle Degeneration
Source: Int J Mol Sci. 2021 Sep 30;22(19):10632. doi: 10.3390/ijms221910632 (PMC8508754; doi:10.3390/ijms221910632)

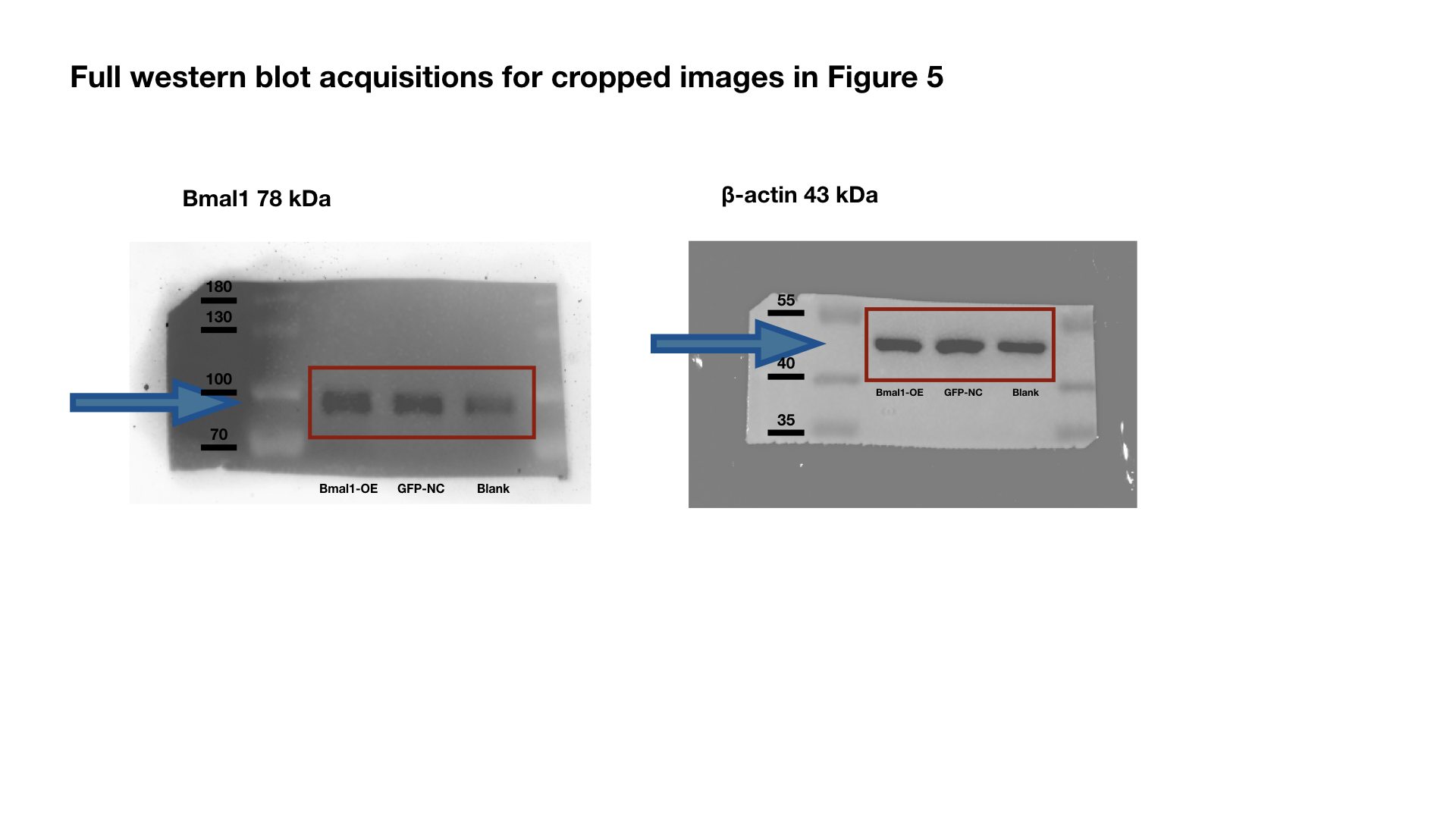

Supplement: Supplementary file 1 [file ijms-22-10632-s001.zip › fig.s2.tiff]

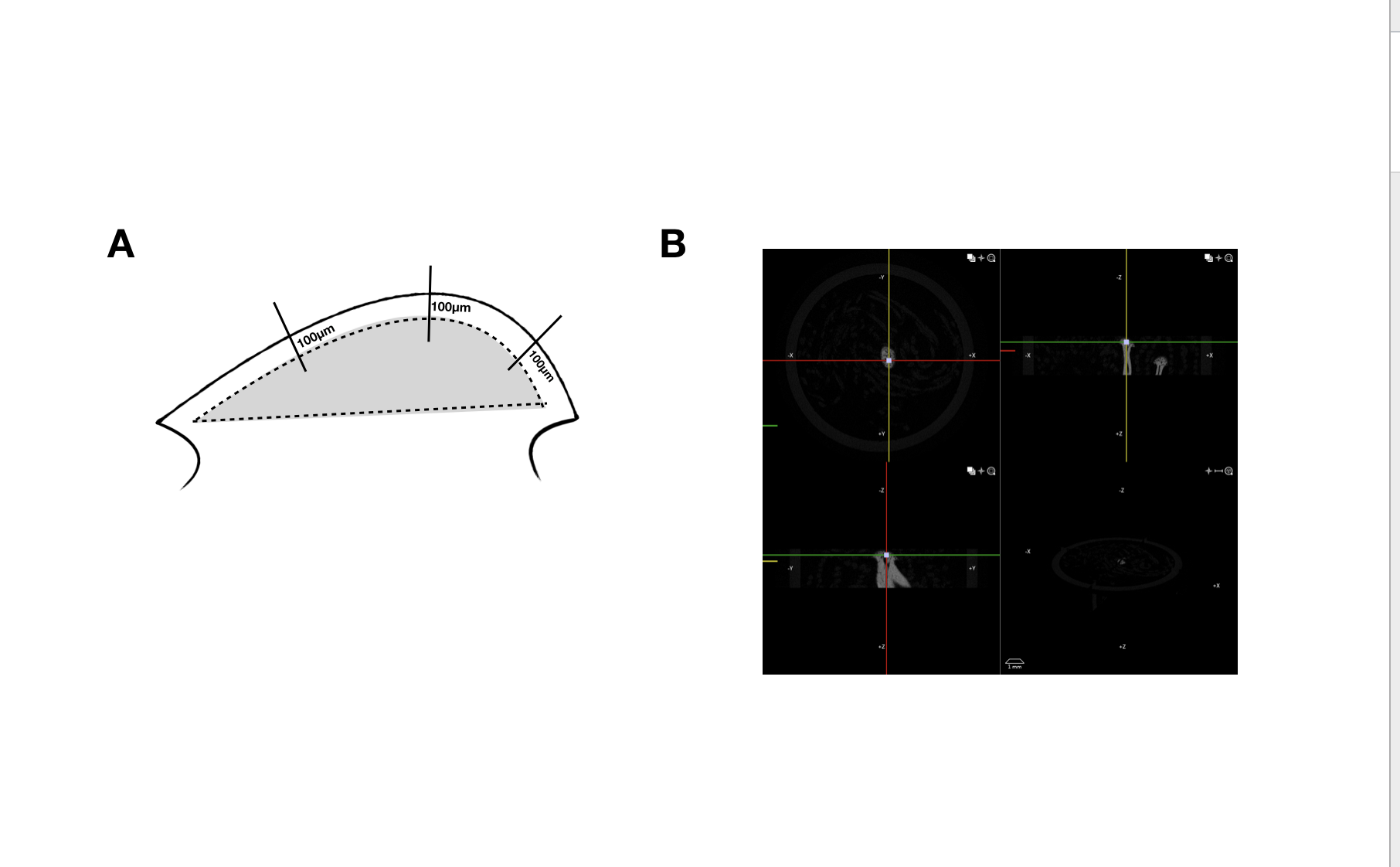

Supplement: Supplementary file 1 [file ijms-22-10632-s001.zip › fig s1.tiff]
